# Reliability of recognition thresholds of sentences in quiet and in noise

**DOI:** 10.1016/S1808-8694(15)31267-2

**Published:** 2015-10-20

**Authors:** Carine Dias de Freitas, Luís Felipe Dias Lopes, Maristela Julio Costa

**Affiliations:** 1 Master studies in Human Communication Disorders under course, UFSM - RS, Clinical Speech therapist and audiologist.; 2 Ph.D. in Production Engineering, Federal University of Santa Catarina - UFSC/SC, Head of the Department of Statistics, Center of Natural and Exact Sciences, UFSM.; 3 Ph.D. in Human Communication Disorders, Speech and Hearing Therapy, Federal University of Sao Paulo - UNIFESP/SP, Audiologist, Joint Professor, School of Speech and Hearing Therapy, UFSM.

**Keywords:** reliability, hearing, speech discrimination, noise

## Abstract

A larger number of research studies has been performed with different people and objectives and have shown that the sentence recognition test in noise is the best instrument to evaluate individuals’ daily communication. However, we believe these tests are not applied so frequently because they require a lot of research to establish the parameters and variables related to their application and interpretation of the results. **Aim**: To check the reliability of the recognition threshold of the sentences in quiet and in noise for a group of young normal listeners. **Study design:** transversal cohort. **Material and Method**: The group comprised 40 subjects, 20 males and 20 females, with ages between 18 and 28 and all of them with normal hearing threshold. First, we applied the Basic Audiological Evaluation and after this, the Sentence Recognition Threshold test in quiet (LRSS) and in noise (LRSR). The sentences and the noise (fixed in 65 dB HL) were presented monoaurally, by earphones through “ascending-descending” strategy. The test and retest were done in different evaluation sessions, with an interval of seven days between them, respecting the same hour of evaluation. **Results**: The results showed strong positive statistically significant correlation between the test and retest of LRSS, both for right ear (r = 0.6107) and left ear (r = 0.5853), as S/N ratio, for right ear (r = 0.5711) and for left ear (r = 0.5867) for the assessed individuals. **Conclusion**: In the end of this study, we concluded that LRSS and S/N ratio obtained from the Portuguese Sentence List Test showed to be highly reliable, with strong positive correlation when compared to the results obtained in different sessions of evaluation in a group of young normal listeners.

## INTRODUCTION

In a basic hearing assessment, even though there are well-established relations between pure tone thresholds and the necessary intensity to understand speech, difficulties to understand speech can only be demonstrated with speech sounds that represent a situation of communication.

In a study about speech perception in noise, it was reported that to assess speech recognition in competitive noise the use of sentences would be better than the use of words, because sentences can better simulate daily situations ^1^, that is, they are closer to spectral characteristics and contexts of everyday conversational speech, at the same time it controls duration and semantic contents of the sentence ^2^.

During the assessments, the skill to understand speech is affected by many factors, such as level of presentation of the material, type of presentation and response and listeners’ characteristics, including language experiences and hearing system conditions. Thus, it emphasizes the importance of performing the tests in noise, given that with the same speech recognition skills in quiet we can obtain extremely different results than in noisy environments ^3^.

In many countries, for over two decades, tests were formed of lists of sentences, given that they are considered to be the best instrument to assess the communication of subjects with hearing disorder complaints 1, 4, 5, 6, 7, 8.

Research studies have been performed in different populations and with different objectives and have demonstrated that the sentence recognition tests in noise are the best instrument to assess the communication of subjects in their daily life. However, it is believed that these tests are still not part of the audiological routine because they require many studies to define parameters and variables related to their application and interpretation of results, in addition to spending more time and unfortunately, owing to lack of awareness of its importance by professionals.

However, we are aware of a larger number of research studies related to this aspect and its gradual inclusion in the batteries that assess auditory disorders.

In Brazil, many studies have been performed to apply the Lists of Sentences in Portuguese (LSP), a test comprising lists of sentences in Brazilian Portuguese, in noise, with speech spectrum that enables the assessment of speech recognition also in the presence of competitive noise ^9^.

The reliability of the test refers to the characteristics that it should have to measure mistakes in a precise and reliable format^10^.

One of the most important characteristics of any speech recognition test is to be able to provide reliability in the repetitive measure of individual and group characteristics. The correlation between this set of results obtained in test-retest is named Coefficient of Correlation, which expresses the level of correspondence that exists between these two applications ^11^. When there are repetitive measures, performed under identical conditions, they result in large differences in test-retest, but the test many not show reliable differences between the populations in the tested conditions ^12^.

Thus, the present study intended to check the reliability of sentence recognition thresholds in quiet and in noise in a group of normal-hearing subjects.

## MATERIAL AND METHOD

The present study was an experiment performed at the Ambulatory of Audiology, Service of Speech and Hearing Pathology (SAF), Federal University of Santa Maria (UFSM), between July to September 2003, after the approval nº 14642 of the Ethics Committee, Office of Health Science Research Projects - CCS, UFSM.

The studied group comprised 40 subjects, 20 male and 20 female subjects, aged between 18 and 28 years (mean age of 22.02 years), all of them with hearing thresholds within the normal ranges ^13^.

The subjects performed a study of Sentence Recognition Thresholds in Quiet (LRSS) and Sentence Recognition Thresholds in Noise (LRSR). Test-retest was performed in different assessment sessions, comprising two sessions to all subjects with an interval of 7 days between them and performed at the same time and period of the day.

Sentence Recognition Thresholds in Quiet (LRSS) and in Noise (LRSR) were obtained by using the tests with List of Sentences in Portuguese (LSP)^9^, which comprised a list of 25 sentences in Portuguese ^14^, seven lists with 10 sentences each ^15^, and noise within the speech spectrum ^16^. Sentences and noise were recorded in CD in independent channels, which enabled the presentation both in quiet and in noise.

During the first study performed with auricular phones ^17^, we detected a difference of 7dB between volume of recording of the two signals in the CD (speech and noise). Next, we performed computer spectrographic analysis of the material recorded in a CD, which confirmed the difference between the two stimuli, demonstrating that the sentences were recorded in mean intensity 7dB below the intensity of noise. For this reason, it is required to subtract the 7dB from the values obtained with the presentation of the sentences, both for LRSS calculation and LRSR calculation, when the VU meter is positioned in zero in both channels, a procedure adopted in this study as well.

Both lists of sentences and competitive noise were presented monoaurally through auricular phones, assessing the ears separately, and the speech assessment in noise presented both ipsilaterally.

Before starting the test with each subject, the output of each channel of the CD was calibrated through the VU meter of the audiometer. The tone of 1kHz present in the same CD channel in which the sentences were recorded, as well as the masking sound present in the other channel, were adjusted to zero.

The presentation of lists of sentences 1A, 1B, 2B, 3B, and 4B, for subjects in the study followed the order below:

a. Presentation of sentences from 1 to 10 in the 1A list, without the presence of competitive noise, on the left, to make the subject familiarized with the test;
b. Presentation of list 1B, without competitive noise, on the right ear;
c. Presentation of list 2B, without competitive noise, on the left ear;
d. Presentation of sentences from 11 to 20 from list 1A, with the presence of competitive noise ipsilaterally on the left to familiarize the subject with the test;
e. Presentation of list 3B, with competitive noise, on the right ear;
f. Presentation of list 4B, with competitive noise, on the left ear.

The test was applied following the “sequential, adaptative or ascending-descending strategy”^18^. The procedure enables the determination of Sentence Recognition Thresholds (LRF), that is, the necessary level for the subject to correctly identify about 50% of the speech stimuli presented, both in quiet (LRSS) and in noise (LRSR). Owing to the technical possibilities of the equipment available for the conduction of this study, we used intervals of sentence presentation of 5 dB and 2.5 dB, respectively.

Upon studying LRSS, the first sentence of each list was presented with intensity of 10 dB above the value found in the LRF study, according to the equipment dial. It corresponded to 3 dB HL (considering the subtraction of 7dB from the speech intensity as observed in the equipment dial), which was enough in the case of subjects with normal hearing. In turn, during the presentation of sentences in competitive noise (LRSR), we used intensity of 70dB in the equipment dial to present the first sentence of each list, which corresponded to 63dB HL in the earphone. Thus, we defined a baseline S/N ratio of - 2 dB, because noise was fixed at 65 dB HL^7^, ^19^. The intensity of presentation of sentences was increased or decreased according to the response of subjects.

Levels of presentation of each sentence were marked down during the assessment. The mean of these values was calculated based on the levels of presentation of each sentence in which there was the first change in response, up to the level of presentation of the last sentence in the list. Finally, we adopted as the procedure to determine the Sentence Recognition Thresholds in Quiet (LRSS) and in Noise (LRSR) the subtraction of 7dB from the presentation value of the sentences, based on the calculation of the mean.

To calculate the Signal/Noise ratio (S/N) we subtracted LRSR from the intensity of the presented noise, in this case, 65dB HL. Thus, the S/N ratio is the difference in dB between the value of LRSR (mean of the intensity of speech in noise) and the competitive noise used.

The measurements were made in acoustically treated booth, using two-channel digital audiometer, brand Fonix, model FA-12, type I, and auricular phones type TDH-39P, brand Telephonics. Sentences and noise were presented by using a Compact Disc Player Digital Toshiba - 4149, coupled to the audiometer above described.

**Figure 1:**
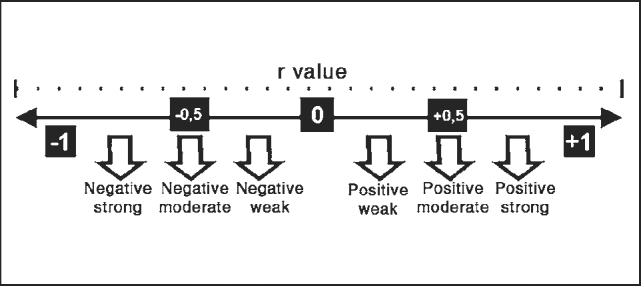
Representative Model of Correlation Coefficient. Barbetta PA. Estatística Aplicada às Ciências Sociais 4ª ed. Florianópolis: Editora da UFSC; 2001.

The results of this study were statistically analyzed using parametric tests. We initially used t Student test to analyze whether there was any statistically significant difference between right and left ears in the test-retest obtained for LRSS and S/N ratio of the assessed subjects. Next, we performed the Analysis of Correlation to check the Correlation Coefficient (r) of the values obtained for test-retest of LRSS and S/N ratio of the assessed subjects, calculating Pearson Correlation Coefficient. This method assumes that the characteristic that is being measured is relatively stable throughout time, at least in the period that separates the two applications. It is also required that the second result is not affected by double exposure ^10^. It means that the closer from positive, the closer to 100% the direct correspondence between both applications will be. Zero coefficient indicates that there is no correlation between what happened in the first and the second application (Figure 1).

The level of rejection of the null hypothesis was fixed at equal or below 5%. The statistically significant results were marked with an asterisk (*).

The results used were based on descriptive analysis of the data, including arithmetic mean, standard deviation, minimum and maximum values from test-retest with LRSS and S/N ratios in the studied group.

## RESULTS

### A. Sentence Recognition thresholds (LRSS) obtained during two assessment sessions (T and R), in young normal hearing subjects (N = 40)

In Table 1 we show the means, standard deviation, Minimum and Maximum values of Sentence Recognition Thresholds in Quiet (LRSS), obtained in the first (T) and second (R) assessment sessions, referring to the right and left ears of 40 studied subjects, as well as the result of the statistical analysis performed with T Student test.

**Table 1:** Comparative analysis between right and left ears of LRSS, obtained in the test-retest of young normal hearing subjects (N = 40).

LRSS (dB NA)						
Variable	N	Mean	Standard deviation	Minimum	Maximum	p-value
Test RE	40	5.82	3.26	-0.75	16.12	p = 0.0163*
Test LE	40	4.14	2.85	-1.58	13.55	
Retest RE	40	4.55	2.93	0.5	11.89	p = 0.4147
Retest LE	40	4.04	2.64	0.5	13.55	

* There was statistically significant difference between the ears - T Student test (p < 0.05). LRSS - Sentence Recognition thresholds in quiet

In Table 2, we present the means, standard deviation, Minimum and Maximum values of LRSS, obtained in the first (T) and second (R) assessments referring to 40 studied subjects including the result of the statistical analysis performed using the Correlation Analysis, named Correlation Coefficient (r).

**Table 2:** Correlation between LRSS obtained in test-retest of young normal hearing subjects (N = 40).

LRSS (dB)							
Variable	N	Mean	Standard deviation		Minimum	Maximum	p-value
Test RE	40	5.82	3.26	-0.75	16.12	0.6107	p = 0.0001*
Retest RE	40	4.55	2.93	0.5	11.89		
Test LE	40	4.14	2.85	-1.58	13.55	0.5853	p = 0.0001*
Retest LE	40	4.04	2.64	0.5	13.55		

* There was statistically significant difference (p < 0.05). LRSS - Sentence Recognition thresholds in quiet

### B. Signal/noise ratio (S/R) obtained in two assessment sessions (T and R), in young normal hearing subjects (N = 40)

In Table 3, we present the means, standard deviations and Minimum and Maximum values of S/N ratios obtained in the first (T) and second (R) assessment sessions, referring to the right and left ears of 40 studied subjects, as well as the result of the statistical analysis performed by t Student test.

**Table 3:** Comparative analysis between right and left ears of S/N ratio obtained in the test-retest of young normal hearing subjects (N=40).

S/N Ratio (dB)						
Variable	N	Mean	Standard deviation	Minimum	Maximum	p-value
Test RE	40	-6.31	1.98	-13.07	-2.56	p = 0.4011
Test LE	40	-6.68	1.87	-11.64	-3.11	
Retest RE	40	-6.88	1.59	-10.13	-4.22	p = 0.4157
Retest LE	40	-7.20	1.93	-12.00	-3.64	

There was no statistically significant difference between the ears - T Student Test (p > 0.05).

In Table 4 we can see the means, standard deviations and Minimum and Maximum values of S/N ratios obtained in the first (T) and second (R) assessment sessions referring to the 40 studied subjects and the result of the statistical analysis performed by the Analysis of Correlation, or Correlation Coefficient (r).

**Table 4:** Correlation between S/N Ratio obtained in the test-retest of young normal hearing subjects (N=40).

S/N RATIO (dB)							
Variable	N	Mean	Standard deviation	Minimum	Maximum	r-value	p-value
Test RE	40	-6.31	1.98	-13.07	-2.56	0.5711	p = 0.0001*
Retest RE	40	-6.88	1.59	-10.13	-4.22		
Test LE	40	-6.68	1.87	-11.64	-3.11	0.5867	p = 0.0001*
Retest LE	40	-7.20	1.93	-12.00	-3.64		

* There was statistically significant difference (p < 0.05).

## DISCUSSION

### A. Sentence Recognition thresholds (LRSS) obtained during the assessment sessions (T and R), in young normal hearing subjects (N = 40)

Upon analyzing the results of LRSS in this study (Table 1), we compared them to other results found with LSP test, using the same methodology used here and they found mean LRSS of 14.32 dB HL^17^; 11.78 dB HL and 12.75 dB HL for RE and 12.03 dB HL and 13.44 dB HL for LE^20^. These values are in agreement with our current results, provided we discount the 7dB previously suggested, as a result of the difference detected between the calibration sound and the competitive noise recorded in the CD.

Similar results were also found in LSP, in young normal hearing subjects, using auricular phones 21, 2, 23. They found mean LRSS of 3.12 dB HL for RE and 4.74 dB HL for LE; 6.58 dB HL for RE and 4.94 dB HL for LE; and mean LRSS of 6.20 dB HL, in the respective studies.

In turn, the analysis performed to compare the results obtained with the first ear (RE) versus the second ear (LE) detected statistically significant difference between them only in the first assessment session (Table 1).

We also detected that the second tested ear presented better results than the first tested ear. Such findings were also seen in other studies 20, 21, 22, 23. Even though the statistical difference has only been found in the first assessment, we could observe that the results of the second tested ear were better in the three assessments.

These results were in agreement with a study performed to investigate the effect of white noise in intelligibility of monosyllable words in normal hearing subjects, in which we detected statistically significant difference concerning the order of tested ears, suggesting that subjects learn during the performance of the assessment ^24^.

Considering that the effect of learning in the procedure may be present during the assessment of speech recognition, we understand the reason why the results were better in the second tested ear. These differences may be observed and should be seen because there are many factors that may interfere in the response of patients in tests that use speech stimulus. Among them we can mention the training of patients during the application of tests ^24^; the effect of learning ^10^; familiarity with words and memory ^25^. In addition, there are physical and linguistic factors related to the listener, including language experiences and language proficiency ^3^.

Thus, we suggest that based on the data observed in the clinical practice, we adopt as test procedure the presentation of five sentences in each ear for familiarization of the patients with the test, trying to minimize the effects of learning in the procedure.

As to Correlation Analysis of LRSS we found strong positive correlation (Figure 1) that was statistically significant between test-retest of LRSS, both for the right ear (r = 0.6107) and the left ear (r = 0.5853) of assessed subjects (Table 2). These findings demonstrate a direct correspondence between the two applications of approximately 61% for the test-retest of the right ear and 58% for the test-retest of the left ear. Thus, we noticed that there was reliability of the instrument and it was referred to the fact that the results were reproduced in different occasions, in which we maintained similar conditions, including the same group of subjects, providing reliable measurements with approximate results, correlating the measures with the same characteristics under the same conditions.

### B. Signal/noise ratio (S/N) obtained during two assessment sessions (T and R) in young normal hearing subjects (N = 40)

S/N ratios found in this study were also compared to other research study performed with LSP test using the same methodology used, and we observed agreement between them. We found for S/N ratio mean of - 6.32 dB HL^17^; - 6.60 dB HL and - 7.87 dB HL for RE, and - 7.68 dB HL and - 7.18 dB HL for LE^20^; - 8.02 dB HL for RE and - 7.41 dB HL for LE^21^; - 5.70 dB HL RE and - 5.94 dB HL for LE^22^; and S/N ratio mean of - 5.29 dB HL^23^.

Next, the analysis to compare the results of the LRSR study obtained between the first (RE) and the second tested ear (LE) did not show statistically significant difference.

However, upon comparing the results obtained between them, we detected that the second assessed ear presented slightly better results in relation to the first tested ear in the three assessments. As reported in quiet measurements, this evidence suggests learning from the procedure during the performance of the assessment^24^.

In this case, it is also believed that the suggested procedure for detection of measurements in quiet is due to be employed in this occasion, that is, presentation of five sentences in competitive noise in each assessed ear to familiarize the patient with the test, to minimize the effect of learning from the procedure. It is recommended that when there are differences greater than 2dB HL between the ears with similar pure tone thresholds, a different list should be presented again on the worst ear ^27^.

Moreover, it is suggested that the examiner selects an initial intensity for the presentation of the first sentence of each list, both in quiet and in noise, which can ensure that the assessed subject is successful in the first sentence and maintains the motivation for the conduction of the whole test, based on the results maintained with the list used for the training.

As to Correlation Analysis of S/N ratio obtained in this study, we detected strong positive correlation (Figure 1) that was statistically significant between the test-retest of LRSR, both for right ear (r = 0.5711) and for left ear (r = 0.5867) of assessed subjects (Table 4). Similarly, these findings evidenced direct correspondence between the two application of about 57% for test-retest on the right ear and 58% for the left ear. Once again, we reached reliable measurements with correlated and close results when we measured again the characteristics of the same subject under the same conditions.

According to Erthal^10^, the application of standardized tests should be performed in a rigorous fashion so that there is no interference of variables in the process. The purpose of the test was to measure existing differences of a specific characteristic among many different subjects, or the behavior of the same subjects in different occasions, inter and intra-subjects differences, respectively.

In addition, an instrument is only valid when the differences of results obtained with the instruments necessarily reflect the real differences between subjects of even within the same subject in different occasions.

## CONCLUSION

Upon completing this study, the critical analysis of the results led us to conclude that:

• The Correlation Coefficient of LRSS was 0.6107 in the test-retest of the right ear and 0.5853 for the test-retest of the left ear, showing statistically significant correlation;
• The Correlation Coefficient of S/N ratios was 0.5711 for the test-retest on the right ear and 0.5867 for the test-retest on the left ear, showing statistically significant correlation;
• LRSS and S/N ratios obtained from the test Lists of Sentences in Portuguese proved to be highly strong, when compared to results obtained in different sessions of assessment in groups of young normal hearing subjects.
